# A monoclonal antibody to Siglec-8 suppresses non-allergic airway inflammation and inhibits IgE-independent mast cell activation

**DOI:** 10.1038/s41385-020-00336-9

**Published:** 2020-08-19

**Authors:** Julia Schanin, Simon Gebremeskel, Wouter Korver, Rustom Falahati, Melina Butuci, Tatt Jhong Haw, Prema M. Nair, Gang Liu, Nicole G. Hansbro, Philip M. Hansbro, Erik Evensen, Emily C. Brock, Alan Xu, Alan Wong, John Leung, Christopher Bebbington, Nenad Tomasevic, Bradford A. Youngblood

**Affiliations:** 1 Allakos, Inc Redwood City, CA 94065 USA; 2 Priority Research Centre for Healthy Lungs, Hunter Medical Research Institute, New Lambton, NSW 2305 and The University of Newcastle, Callaghan, NSW 2208 Australia; 3 Centre for Inflammation, Centenary Institute, Sydney, NSW 2050, and University of Technology Sydney, School of Life Sciences, Faculty of Science, Ultimo, NSW 2007 Australia; 4 Basis Bioscience, LLC, Foster City, CA 94065 USA

## Abstract

In addition to their well characterized role in mediating IgE-dependent allergic diseases, aberrant accumulation and activation of mast cells (MCs) is associated with many non-allergic inflammatory diseases, whereby their activation is likely triggered by non-IgE stimuli (e.g., IL-33). Siglec-8 is an inhibitory receptor expressed on MCs and eosinophils that has been shown to inhibit IgE-mediated MC responses and reduce allergic inflammation upon ligation with a monoclonal antibody (mAb). Herein, we evaluated the effects of an anti-Siglec-8 mAb (anti-S8) in non-allergic disease models of experimental cigarette-smoke-induced chronic obstructive pulmonary disease and bleomycin-induced lung injury in Siglec-8 transgenic mice. Therapeutic treatment with anti-S8 inhibited MC activation and reduced recruitment of immune cells, airway inflammation, and lung fibrosis. Similarly, using a model of MC-dependent, IL-33-induced inflammation, anti-S8 treatment suppressed neutrophil influx, and cytokine production through MC inhibition. Transcriptomic profiling of MCs further demonstrated anti-S8-mediated downregulation of MC signaling pathways induced by IL-33, including TNF signaling via NF-κB. Collectively, these findings demonstrate that ligating Siglec-8 with an antibody reduces non-allergic inflammation and inhibits IgE-independent MC activation, supporting the evaluation of an anti-Siglec-8 mAb as a therapeutic approach in both allergic and non-allergic inflammatory diseases in which MCs play a role.

## Introduction

Mast cells (MCs) are tissue-resident cells that have broad roles in regulating acute and chronic tissue inflammation in a variety of allergic, proliferative, and inflammatory diseases. Historically, MCs have been associated with allergic inflammation, during which their activation plays a critical role in driving type-2 inflammatory diseases, such as eosinophilic asthma, atopic dermatitis, and eosinophilic gastrointestinal diseases.^1^ Activation of MCs by crosslinking the FcεRI via IgE induces rapid degranulation and the release of preformed mediators, such as histamine, tumor necrosis factor (TNF), and proteases, as well as the subsequent release of de novo synthesized lipid mediators, cytokines, and chemokines. Many of these substances attract or activate other immune, epithelial, neuronal, and stromal cells that contribute to acute and chronic allergic responses, such as vasodilation, plasma extravasation, smooth muscle contraction, tissue eosinophilia, and remodeling.^1^ In addition to allergic inflammation, MCs have been implicated in non-allergic diseases, such as inflammatory bowel disease, chronic obstructive pulmonary disease (COPD), type-2 low asthma, idiopathic pulmonary fibrosis (IPF), and psoriasis.^2–4^ In these disease settings, MCs are likely activated by inflammatory mediators, such as cytokines, Toll-like receptor (TLR) ligands, and neuropeptides.^2,5^

The alarmin cytokine interleukin (IL)−33 is one such mediator that can directly activate MCs independent of IgE to promote non-allergic inflammation, including neutrophil infiltration, tissue remodeling, and fibrosis.^6^ IL-33 is released from cells following tissue damage, stress, or cell death, and exerts its proinflammatory biological functions through the ST2L receptor, which is present on a subset of immune cells, including MCs and eosinophils. Consistent with its role in tissue damage and fibrosis, increased expression of IL-33 and ST2L have been observed in chronic non-allergic airway diseases, such as COPD and IPF.^7,8^ COPD and IPF are debilitating disorders of the lung characterized by chronic airway inflammation and fibrosis that compromise tissue architecture, lung function, and gas exchange. In addition to increased expression of IL-33/ST2L, MC numbers are elevated in COPD and IPF patient lung tissue.^3,4,7^ Experimental animal models of COPD and IPF further support the pathogenic role of MCs and IL-33/ST2L.^3,4,9,10^ Indeed, mice deficient in MC tryptase have significantly reduced airway inflammation, epithelial thickening, and emphysematous damage in a cigarette smoke (CS)-induced experimental COPD model.^3,4^ In addition, bleomycin (BLM)-induced lung fibrosis and inflammation are attenuated in MC- or ST2-deficient mice or with administration of a neutralizing IL-33 antibody.^9,10^ Collectively, these data suggest that IL-33-mediated MC activation may play a role in the pathogenesis of chronic non-allergic airway diseases and provide a rationale for targeting MCs as a potential therapeutic strategy.

Sialic-acid-binding immunoglobulin-like lectin (Siglec)−8 is a cell surface receptor that has emerged as a promising therapeutic target for the treatment of allergic and inflammatory diseases. Siglec-8 is an inhibitory receptor that is found selectively on human MCs and eosinophils. Binding of a monoclonal antibody (mAb) to Siglec-8 has been shown to induce death of cytokine-primed eosinophils and inhibit IgE-mediated mast cell activation.^11–14^ In addition, treatment with an anti-Siglec-8 mAb (anti-S8) has been shown to significantly reduce allergic gastrointestinal inflammation in a novel Siglec-8 transgenic (tg) mouse model in which the human Siglec-8 transgene is constitutively expressed on murine mast cells, eosinophils, and to a lesser extent, basophils.^15^ Therapeutic modulation of MCs via targeting of Siglec-8 has also been evaluated clinically with AK002, a humanized, nonfucosylated IgG1 anti–Siglec-8 mAb that depletes eosinophils via antibody-dependent cellular cytotoxicity (ADCC) and inhibits IgE-dependent MC activation.^11^ Consistent with the critical role that MCs play in allergic diseases and the inhibitory activity mediated by Siglec-8, AK002 has demonstrated significant symptomatic improvement in allergic diseases such as eosinophilic gastritis/enteritis, chronic urticaria, and severe allergic conjunctivitis.^16–18^

While Siglec-8 engagement has been shown to inhibit IgE-driven mast cell activation and allergic inflammation, such effects have not yet been evaluated in non-allergic inflammation. In this study, we demonstrate that anti-S8 treatment inhibits MC activation and reduces immune cell infiltration, airway inflammation, and lung fibrosis in non-allergic disease models of COPD and acute and chronic BLM-induced lung fibrosis. We also provide evidence that anti-S8 treatment inhibits IL-33-mediated MC activation and neutrophil influx by globally modulating the MC transcriptome, using a model of MC-driven, IL-33-induced inflammation. Lastly, we show that anti-S8 reduces human neutrophil migration mediated by IL-33-activated human peripheral blood derived MCs (hMCs). Collectively, our findings demonstrate that anti-S8 treatment reduces acute and chronic non-allergic inflammation and inhibits IgE-independent MC activation, supporting clinical evaluation of anti-Siglec-8 mAbs, such as AK002 as a therapeutic approach for both allergic and non-allergic inflammatory diseases.

## Results

### Therapeutic anti-Siglec-8 mAb treatment reduces chronic inflammation in CS-induced experimental COPD

**Fig. 1:**
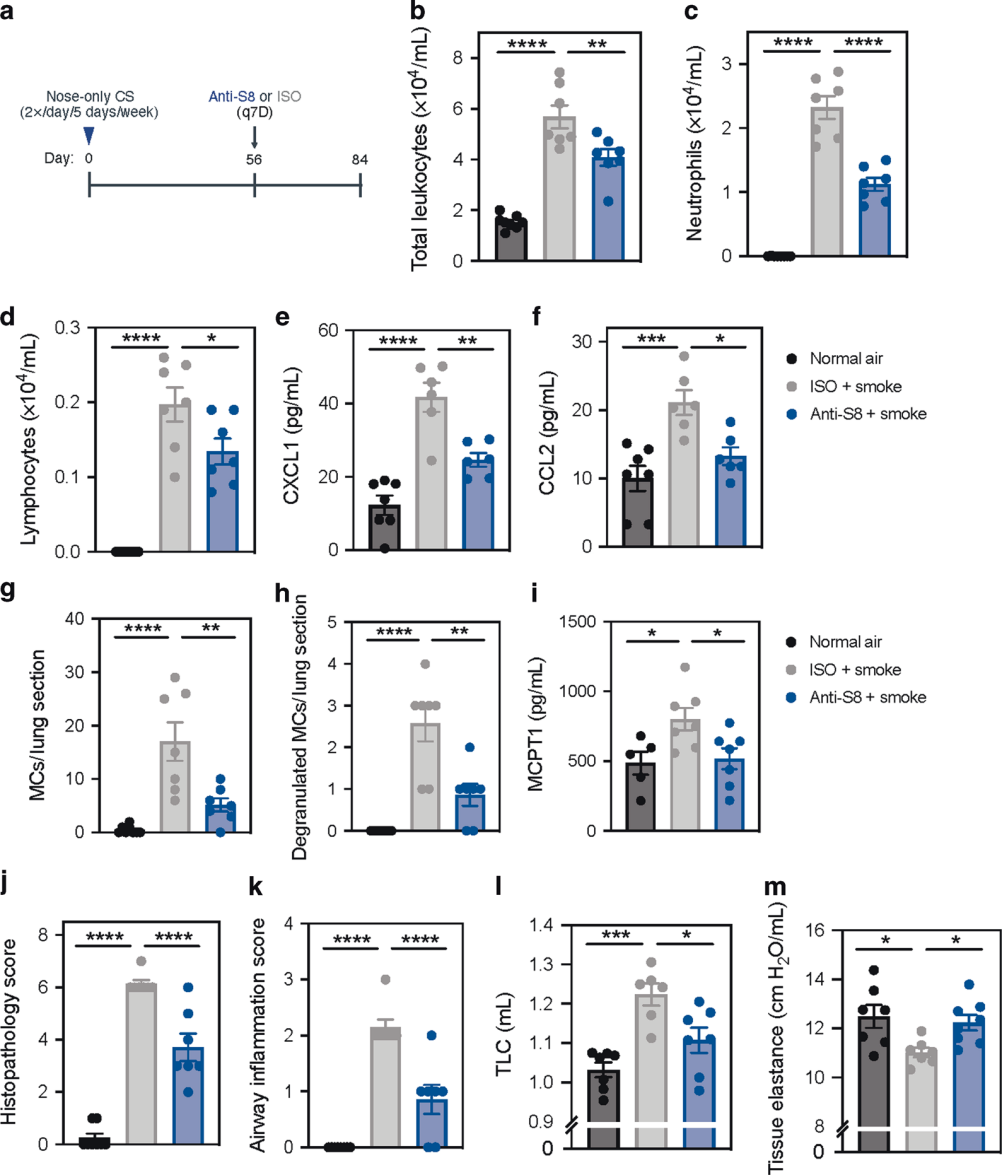
Therapeutic treatment with Siglec-8 mAb reduces CS-induced airway inflammation and improves lung function in experimental COPD. **a** Schematic of experimental COPD model. **b** Total leukocytes, **c** neutrophils, **d** lymphocytes, **e** CXCL1 levels, **f** and CCL2 levels in BAL fluid from mice exposed to normal air (black), ISO + smoke (gray) or anti-S8 + smoke (blue). Enumeration of **g** absolute lung MCs and **h** degranulating MCs by chloroacetate esterase staining, and **i** quantification of MCPT1 in serum of mice exposed to normal air (black), ISO + smoke (gray) or anti-S8 + smoke (blue). **j**, **k** Total histopathology score in lung sections or specifically in the airways. **l**, **m** Total lung capacity and tissue elastance lung function parameters. Data are plotted as mean ± SEM (6–8 mice/group) *P < 0.05; **P < 0.01; ***P < 0.001; ****P < 0.0001 by one-way ANOVA with Tukey’s multiple-comparisons test. BAL bronchoalveolar lavage, q7D dosed every 7 days, COPD chronic obstructive pulmonary disease, MCPT mast cell protease, TLC total lung capacity, ISO isotype control.

To evaluate the inhibitory activity of anti-S8 in chronic non-allergic inflammation, we used a model of CS-induced experimental COPD that has previously been shown to be partially MC-dependent and have several hallmark features of human disease.^3,4^ COPD was induced in Siglec-8 tg mice through nose-only exposure of CS for 12 weeks. Starting in week 8, when key features of COPD have developed,^3,4^ mice were therapeutically dosed weekly with either anti-S8 or isotype-matched control mAb until week 12 (Fig. 1a). Mice were sacrificed and evaluated for markers of inflammation and features of human COPD. To evaluate if anti-S8 treatment decreased airway inflammation induced by CS, we assessed pulmonary inflammation in bronchoalveolar lavage (BAL) fluid by staining and differential enumeration of inflammatory cells at week 12. Compared to mice exposed to normal air, CS-exposed mice had significantly increased numbers of total leukocytes, neutrophils, lymphocytes, and macrophages in BAL (Figs. 1b–d and S1A). Therapeutic treatment with anti-S8 significantly decreased total leukocytes induced by CS exposure, including reductions in neutrophils and lymphocytes, but not macrophages, compared to isotype control-treated mice. In addition, smoke exposure increased the levels of chemokines associated with immune cell infiltration, such as CXCL1 and CCL2, which were significantly reduced in anti-S8-treated mice compared to isotype control-treated mice (Fig. 1e, f). Consistent with a role for MCs in experimental COPD, CS-exposed mice had increased numbers of MCs as well as degranulating MCs in the lungs that were significantly reduced in anti-S8 treated mice (Figs. 1g, h and S1B). In addition, the level of MC protease-1 (MCPT1) was significantly elevated in mice exposed to CS and decreased with anti-S8 treatment (Fig. 1i). Anti-S8 treatment also significantly reduced histopathologic, airway, parenchymal, and vascular inflammation scores induced by chronic CS exposure compared to isotype control mAb-treated mice (Fig. 1j, k and S1C–E). These data demonstrate that anti-S8-treatment reduces MC accumulation and degranulation and suppresses non-allergic chronic inflammation induced by CS.

We next investigated how the suppression of inflammation impacted lung function in mice treated with anti-S8 or isotype control mAb. Compared to mice exposed to normal air, CS exposure significantly increased total lung capacity (TLC) that was significantly reduced in anti-S8-treated mice (Fig. 1l). CS exposure also significantly decreased tissue elastance (Fig. 1m) and forced expiratory volume in 100 milliseconds (FEV_100_)/forced vital capacity (FVC) ratio (representative of FEV_1_/FVC ratio in humans), both of which were significantly improved in mice treated therapeutically with anti-S8 (Figs. 1m and S1F). These data demonstrate that therapeutic treatment with anti-S8 significantly reduces chronic airway inflammation induced by long-term smoke exposure and protects against CS-induced changes in lung function.

### Anti-Siglec-8 mAb treatment decreases acute and chronic BLM-induced lung inflammation and fibrosis

The reduction in lung inflammation and tissue damage observed in experimental COPD suggested that anti-S8 treatment may have the potential to decrease tissue remodeling in non-allergic disease settings. To investigate this further, we evaluated the activity of anti-S8 in acute and chronic BLM-induced lung fibrosis models.

**Fig. 2:**
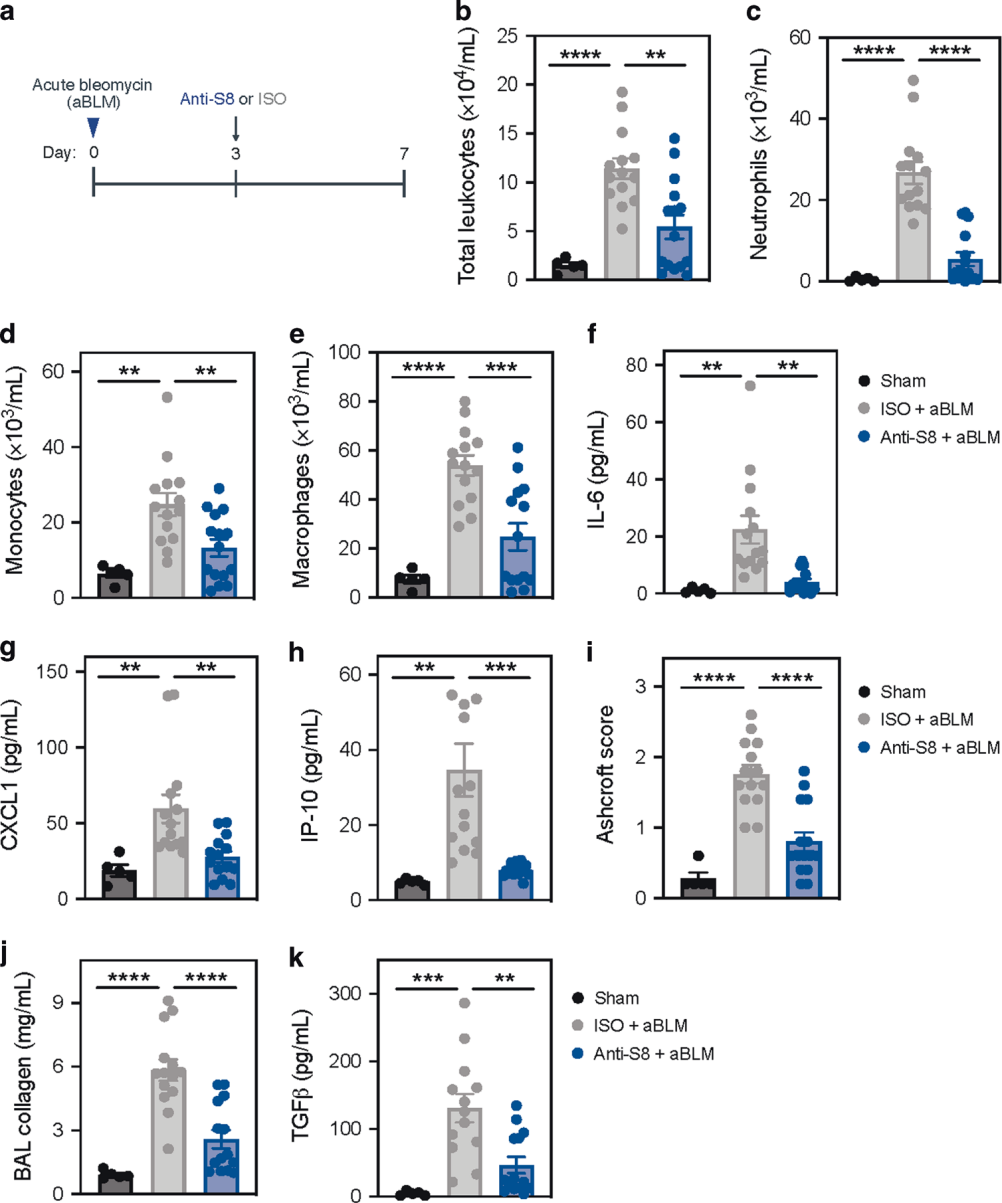
Siglec-8 mAb treatment reduces acute BLM-induced lung inflammation and fibrosis. **a** Schematic of acute BLM-induced lung fibrosis model. **b** Total leukocytes, **c** Neutrophils, **d** monocytes, **e** macrophages, **f** IL-6, **g** CXCL1, and **h** IP-10 in BAL fluid of sham (black), ISO + acute BLM (gray), or anti-S8 + acute BLM (blue) treated mice. **i** Ashcroft scores of lung fibrosis and BAL fluid levels of **j** collagen, and **k** TGFβ in sham (black), ISO + acute BLM (gray), or anti-S8 + acute BLM (blue) treated mice. Data are plotted as mean ± SEM (10–14 mice/group) and are representative of at least three experiments. **P < 0.01; ***P < 0.001; ****P < 0.0001 by one-way ANOVA with Tukey’s multiple-comparisons test. BAL bronchoalveolar lavage, BLM bleomycin, ISO isotype control.

To evaluate the activity of anti-S8 in pulmonary fibrosis, we used an acute model of BLM-induced lung injury. In this model, lung inflammation peaks at ~3 days post intratracheal BLM administration. Therefore we dosed mice at this time point with a single injection of anti-S8 mAb (Fig. 2a). Compared to sham-treated mice, BLM administration significantly increased airway inflammation as evidenced by infiltration of leukocytes into the BAL fluid, including neutrophils, monocytes, and macrophages, whereas eosinophils were not detected (Fig. 2b–e, data not shown). In addition, intratracheal BLM administration increased cytokines and chemokines associated with immune cell recruitment in the BAL fluid (Fig. 2f–h). One dose of anti-S8 significantly suppressed BLM-induced infiltration of neutrophils, monocytes, and macrophages as well as induction of cytokines and chemokines associated with immune cell recruitment in BAL fluid, such as IL-6, CXCL1, and IP-10 (Fig. 2b–h). Treatment with anti-S8 also resulted in a significant reduction in lung fibrosis as assessed by Ashcroft score and decreased levels of collagen and TGFβ in BAL (Fig. 2i–k). These data demonstrate that therapeutic treatment with anti-S8 reduces lung inflammation and fibrosis in an acute model of BLM-mediated lung injury.

**Fig. 3:**
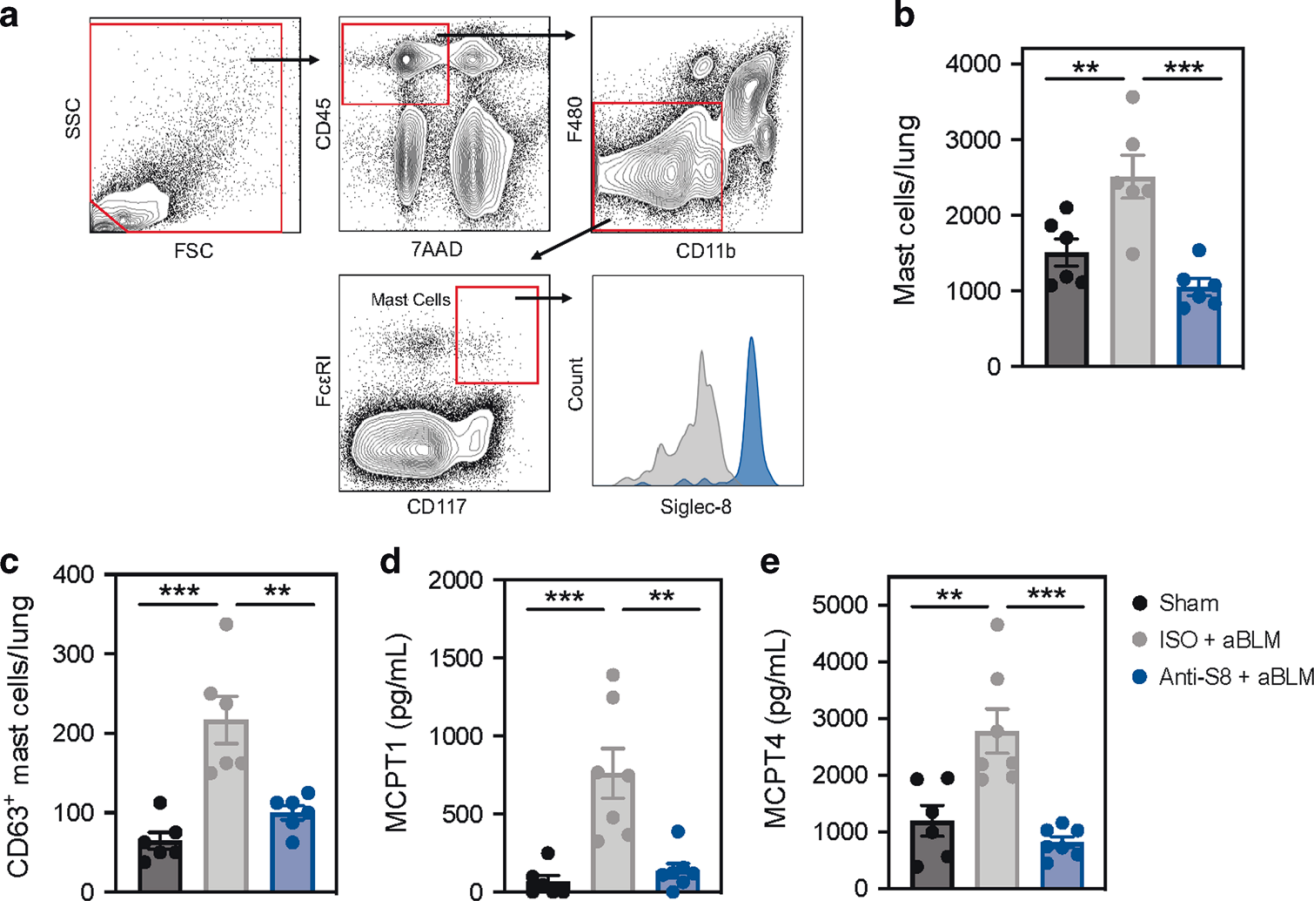
Siglec-8 mAb treatment decreases MC activity in acute BLM-mediated lung injury. **a** Flow cytometry gating strategy to identify MCs in lungs from Siglec-8 tg mice stained with an anti-Siglec-8 mAb (blue) or FMO (gray). **b** Absolute MCs and **c** CD63^+^ MCs per lung determined by flow cytometry in sham (black), ISO + acute BLM (gray), or anti-S8 + acute BLM (blue) treated mice. **d**, **e** Levels of MCPT1 and MCPT4 from overnight ex vivo lung culture in sham (black), ISO + acute BLM (gray), or anti-S8 + acute BLM (blue) treated mice. Data are plotted as mean ± SEM (six mice/group). **P < 0.01; ***P < 0.001 by one-way ANOVA with Tukey’s multiple-comparisons test. BLM bleomycin, FMO fluorescence minus one, ISO isotype control, MCPT mast cell protease.

To assess if the decrease in inflammation and fibrosis in mice treated with anti-S8 was associated with reduced MC activity, we quantified lung MC numbers and activation state by flow cytometry and MCPT1 and MCPT4 levels in ex vivo cultured lung tissue after acute BLM-injury. Lung MCs were identified as CD45^+^ 7AAD^−^ F480^−^ CD11b^−^ CD117^+^ FceRI^+^ and expressed Siglec-8 consistent with previous studies^15^ (Fig. 3a). Mice exposed to BLM had increased numbers of MCs and activated MCs as assessed by the degranulation marker CD63 (Fig. 3b, c). MCPT1 and MCPT4 levels were also significantly increased in lungs from BLM- compared to sham-exposed mice (Fig. 3d, e). Treatment with anti-S8 significantly decreased MC numbers, activation state, and tissue levels of MCPT1 and MCPT4, indicative of MC inhibition (Fig. 3b–e). These data suggest the reduction of inflammation and fibrosis in anti-S8 treated mice is associated with MC inhibition.

While single intratracheal administration of BLM induces acute inflammation and lung fibrosis, it does not lead to cumulative tissue damage and chronic inflammation. To evaluate anti-S8 treatment in a chronic model of lung inflammation and fibrosis, we adapted a repetitive subcutaneous model where BLM is administered every 2 days for 30 days.^19^ Anti-S8 was therapeutically dosed on day 7 and weekly through day 28 (Fig. S2A). Chronic subcutaneous injections of BLM significantly increased neutrophils and macrophages in BAL fluid that were reduced in anti-S8-treated mice (Fig. S2B, C). In addition, anti-S8 treatment significantly reduced lung fibrosis as assessed by histology, Ashcroft score, and lung tissue hydroxyproline levels (Fig. S2D, E). Collectively, these data show that anti-S8 treatment inhibits MC activity and reduces BLM-mediated inflammation and lung fibrosis in acute and chronic non-allergic disease settings.

### Siglec-8 mAb treatment reduces IL-33-mediated neutrophil and monocyte infiltration through MC inhibition

We next determined whether anti-S8 treatment could directly inhibit non-IgE-mediated MC activation along with suppressing non-allergic MC disease manifestations. To evaluate the inhibitory activity of anti-S8 mAb in non-IgE-driven inflammation, we adapted a mechanistic model of MC-dependent, IL-33-mediated inflammation.^6^ In this model, intraperitoneal administration of IL-33 induces a rapid neutrophil influx into the peritoneal cavity that was shown to be completely dependent on peritoneal MCs and ST2L using MC and ST2-deficient mice.^6^ To confirm that IL-33 administration could induce neutrophil recruitment in Siglec-8 tg mice, we analyzed peritoneal immune cells 3 h after IL-33 injection. Consistent with previous findings, IL-33 dose-dependently induced a significant neutrophil influx in the peritoneal cavity that was not associated with a change in MC numbers in Siglec-8 tg mice (Fig. S3A, B). IL-33 administration also dose-dependently induced eosinophil and monocyte infiltration in the peritoneal cavity (Fig. S3C, D), whereas other immune cells, such as macrophages and lymphocytes remained unchanged (data not shown).

**Fig. 4:**
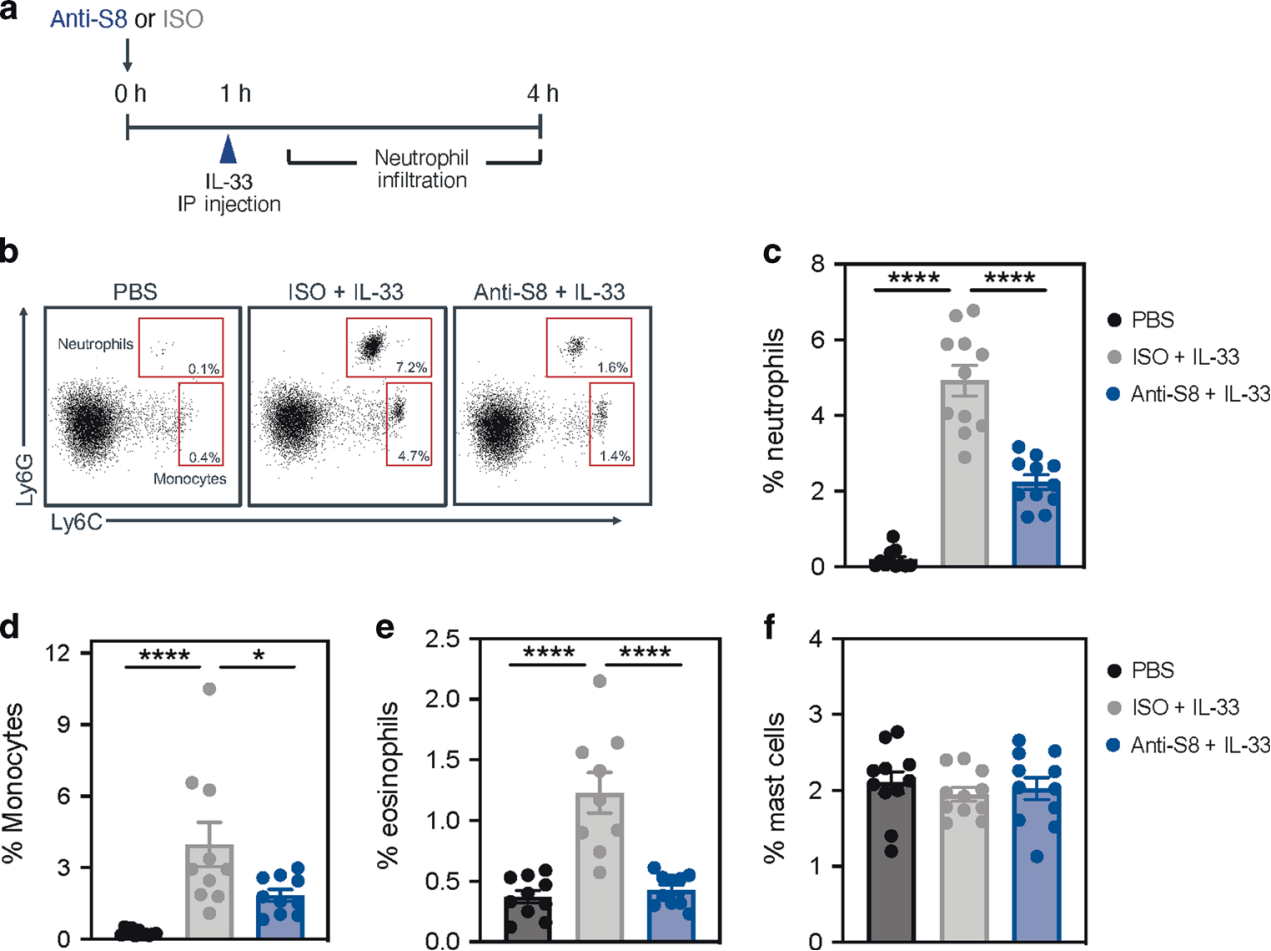
Siglec-8 mAb treatment reduces IL-33-driven leukocyte infiltration in the peritoneal cavity through MC inhibition. **a** Schematic of IL-33-induced inflammation model with Siglec-8 or isotype control mAb treatment. **b** Representative flow cytometry dot plots of neutrophils and monocytes in the peritoneal cavity at 4 h in PBS, ISO + IL-33, and anti-S8 + IL-33 treated groups. Percentage of **c** neutrophils, **d** monocytes, **e** eosinophils, and **f** mast cells in the peritoneal cavity of mice treated with PBS (black), ISO + IL-33 (gray), or anti-S8 + IL-33 (blue). The percentage of immune cells was derived from the CD45^+^ viable cell population. Data are plotted as mean ± SEM (11–12 mice/group) and are representative of at least four experiments. *P < 0.05; ****P < 0.0001 by one-way ANOVA with Tukey’s multiple-comparisons test. IP intraperitoneal, ISO isotype control, PBS phosphate-buffered saline.

We next evaluated IL-33-mediated leukocyte infiltration in the peritoneal cavity following pre-dosing with anti-S8 or isotype control mAb 1 h before IL-33 administration (Fig. 4a). As was observed previously, IL-33 administration induced a significant influx of neutrophils, monocytes, and eosinophils in the peritoneal cavity compared to phosphate-buffered saline (PBS)-administered control mice (Fig. 4b–e). Anti-S8 treatment significantly reduced IL-33-induced neutrophil influx in the peritoneal cavity compared to isotype control-treated mice. Similarly, anti-S8 treatment significantly decreased IL-33-induced monocyte and eosinophil infiltration. Consistent with previous findings, anti-S8 treatment did not reduce mast cell numbers (Fig. 4f), suggesting the reduction in IL-33-driven immune cell infiltration in the peritoneal cavity is associated with mast cell inhibition, not apoptosis or ADCC.

To determine whether the suppression of IL-33-driven inflammation by anti-S8 treatment was solely due to Siglec-8-mediated MC inhibition, we depleted eosinophils in Siglec-8 tg mice using an anti-CCR3 antibody. Anti-CCR3 mAb administration significantly reduced eosinophils in the blood and spleen, consistent with previous studies (Fig. S3E–H).^20^ Next, we depleted eosinophils with anti-CCR3 and administered IL-33 intraperitoneally to ensure IL-33-dependent neutrophil recruitment was not dependent on eosinophils. Eosinophil depletion did not decrease IL-33-induced neutrophil influx compared to mice with eosinophils (Fig. S3I). These data suggest that eosinophils do not contribute to IL-33-mediated neutrophil influx and strongly suggest that anti-S8 treatment significantly reduces acute IL-33-mediated leukocyte infiltration in the peritoneal cavity by inhibiting MCs.

### Siglec-8 mAb treatment decreases local and systemic cytokines/chemokines induced by IL-33

**Fig. 5:**
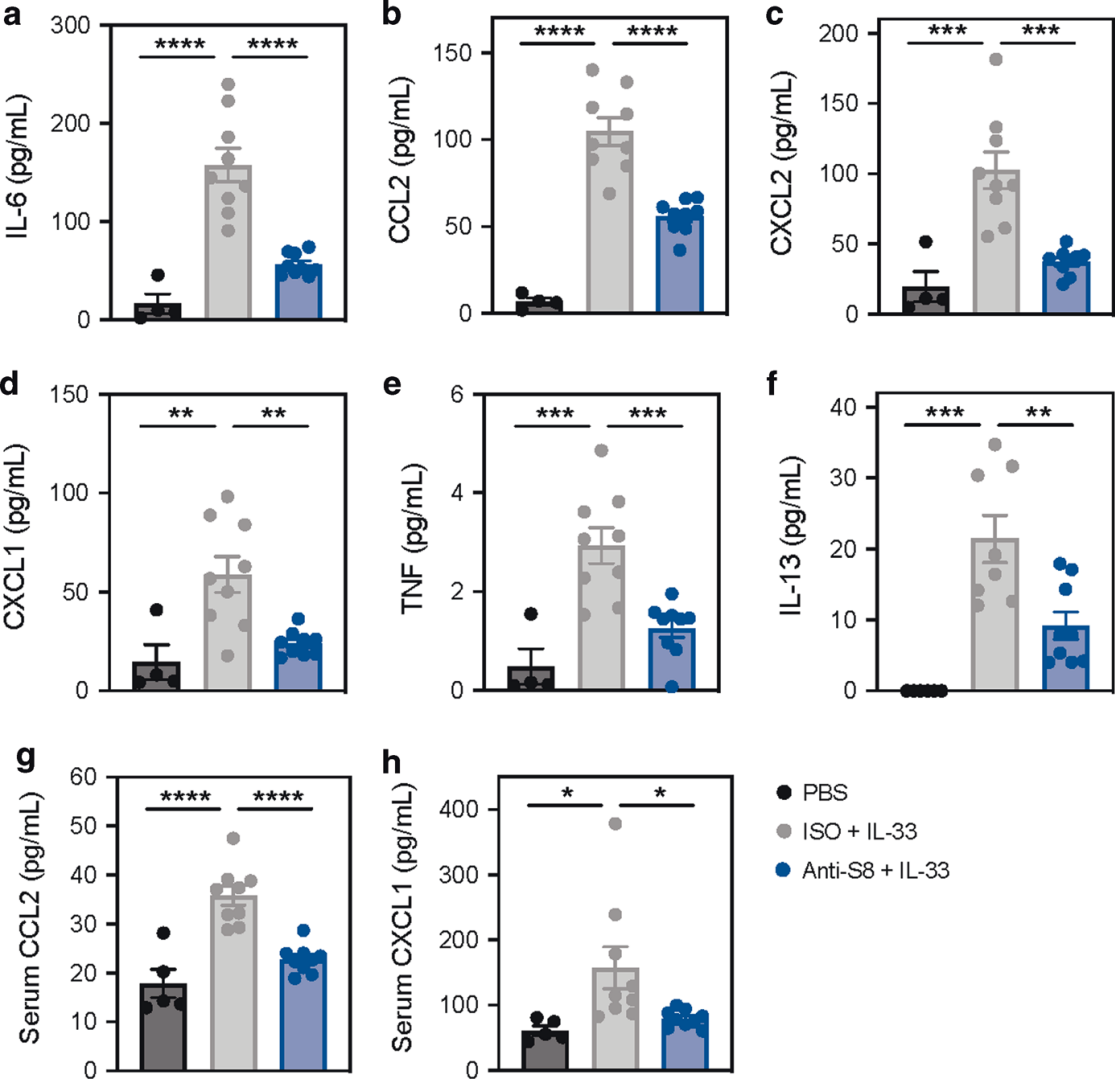
Siglec-8 mAb treatment decreases local and systemic cytokines and chemokines induced by IL-33. **a**–**f** Levels of IL-6, CCL2, CXCL2, CXCL1, TNF, and IL-13 in the peritoneal cavity and (G-H) CCL2 and CXCL1 in the serum of mice treated with PBS (black), ISO + IL-33 (gray) or anti-S8 + IL-33 (blue). Data are plotted as mean ± SEM (9–10 mice/group) and are representative of at least four experiments. *P < 0.05; **P < 0.01; ***P < 0.001; ****P < 0.0001 by one-way ANOVA with Tukey’s multiple-comparisons test. ISO isotype control, PBS phosphate-buffered saline.

Unlike IgE-mediated activation of MCs, IL-33 does not stimulate degranulation, but instead, induces the secretion of cytokines and chemokines from MCs.^21^ We next evaluated if the reduction in IL-33-driven leukocyte infiltration observed in anti-S8-treated mice was associated with a decrease in mediators that recruit these immune cells. Consistent with the influx of immune cells, intraperitoneal administration of IL-33 significantly increased the levels of cytokines and chemokines associated with neutrophil, monocyte, and eosinophil infiltration in the peritoneal cavity, including IL-6, CCL2, CXCL2, CXCL1, TNF, IL-13, CCL3, and CCL4 (Figs. 5a–f and S4A, B). Treatment with anti-S8 significantly decreased IL-33-induced cytokines and chemokines compared to isotype control mAb mice. Since many of these mediators can be produced by multiple types of immune cells, we cultured peritoneal MCs in vitro and found that stimulation with IL-33 produced a similar profile of cytokines and chemokines as in peritoneal lavage in vivo (Fig. S4C). These data are consistent with ST2L (IL-33R) being mainly found on mast cells in the peritoneal cavity, (Fig. S4D) and suggest that MCs are the initial source of these mediators upon IL-33 administration.

Having established peritoneal levels of these IL-33-induced cytokines and chemokines, we next evaluated IL-33-induced systemic effects by analyzing mediators in serum. IL-33 administration also increased serum levels of cytokines and chemokines that were significantly reduced in anti-S8 treated mice, including the monocyte- and neutrophil-attracting chemokines, CCL2 and CXCL1 (Fig. 5g, h). These data demonstrate that anti-S8 treatment significantly reduces local and systemic IL-33-induced cytokines and chemokines and suggest that MCs are a major target cell of IL-33.

### Siglec-8 mAb treatment modulates the transcriptome of MCs to suppress IL-33-mediated inflammation

**Fig. 6:**
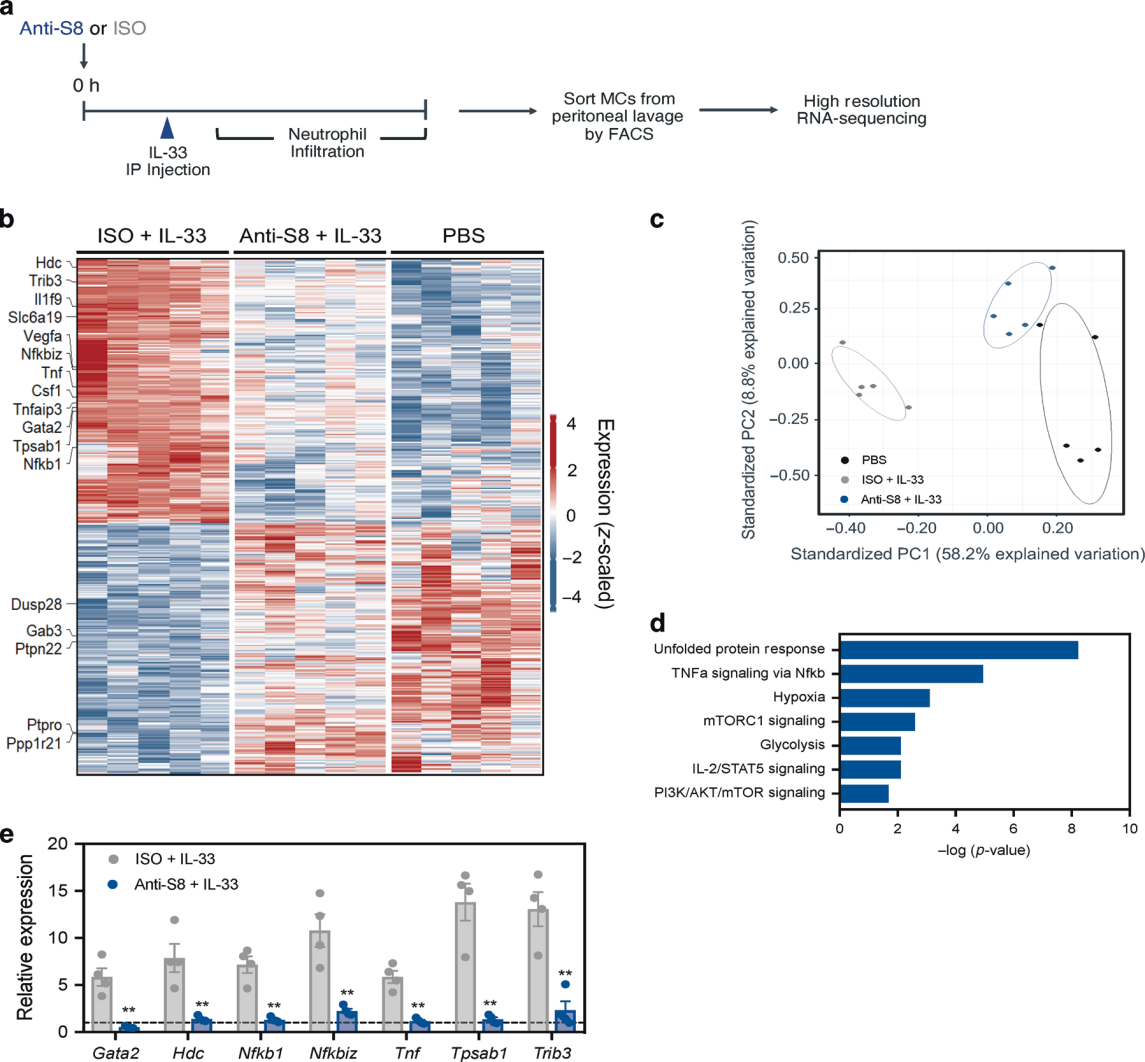
Siglec-8 mAb negatively modulates the IL-33-activated MC transcriptome in vivo. **a** Schematic of in vivo RNA-seq experimental design. **b** Heatmap of the top differentially expressed genes (absolute log2Fold >0.4, unadjusted *p* < 0.05) from FACS-sorted peritoneal MCs after in vivo administration of IL-33 + ISO (left), IL-33  + anti-S8 (middle), or PBS (right). Each column represents transcriptome data from individual mice (*n* = 5/group). **c** Principal component analysis of genes displayed in the heatmap from ISO + IL-33 (gray), anti-S8 + IL-33 (blue), or PBS control mice (black). **d** Hallmark enrichment analysis of anti-S8 downregulated genes that were activated with IL-33. **e** Confirmatory qPCR analysis of genes identified in RNA-seq dataset from FACS-sorted peritoneal MCs treated with IL-33 + ISO (gray) or anti-S8 (blue). Dashed line represents normalized gene expression of PBS control peritoneal MCs. For panel **e**, data are plotted as mean ± SEM (*n* = 4/group; three mice pooled/sample). **P < 0.01 by Mann–Whitney *U* test. FACS fluorescence-activated cell sorting, ISO isotype control, PBS phosphate-buffered saline, SEM standard error of the mean.

To better characterize anti-S8-induced inhibition of IL-33-activated MCs and to gain insight into the mechanism of MC inhibition, we assessed the transcriptional profile of isolated peritoneal MCs after IL-33 administration in vivo. Following the induction of IL-33-driven inflammation, peritoneal MCs were FACS-sorted from individual mice based on CD117 and FcεRI expression and subjected to high resolution gene-expression analysis by RNA-seq (Figs. 6a and S5A). Consistent with a highly enriched MC population, we found a significant correlation between MC-specific genes in our dataset with the previously published transcriptional signature of tissue-resident MCs, including *Tpsab1, Tpsab2, Cma1*, and *Mrgprb2* (Fig. S5B).^22^ To identify gene expression modulated by IL-33 administration, we performed a comparison of mice treated with IL-33 vs. PBS control for 3 h. As expected, IL-33 treatment significantly changed the transcriptional profile of peritoneal MCs (Fig. S5C). Using enrichment analysis, we analyzed differentially expressed genes to identify hallmark gene sets overrepresented in MCs activated by IL-33. These gene sets participated in mTORC signaling, TNF signaling through NF-κB, unfolded protein response, and IL-6 *via* STAT3 signaling (Fig. S5D), consistent with previously published data.^23^ These data demonstrate that IL-33 directly activates and modulates the transcriptome of peritoneal MCs in vivo to drive inflammation.

Next, we assessed the effect of anti-S8 treatment on IL-33-induced changes in the MC transcriptome by comparing mice treated with IL-33 and isotype control mAb *versus* IL-33 and anti-S8 (Fig. 6a). We identified 498 differentially expressed genes that were up- or downregulated upon IL-33 administration and significantly changed after anti-S8 treatment (Fig. 6b). Many of these genes were associated with MC activity and function, such as *Hdc, Tnf, Slc6a19, Nfkb1, Il1f9*, and *Tpsab1*. Principal component analysis further highlighted the differences between IL-33-activated MCs and anti-S8-inhibited MCs and the similarities between anti-S8-inhibited and PBS control MCs (Fig. 6c). To analyze pathways associated with anti-S8-mediated inhibition of IL-33-activated MCs, we used enrichment analysis to identify top differentially expressed hallmark gene sets that were overrepresented among the genes upregulated in IL-33-activated MCs and downregulated after anti-S8 treatment. Anti-S8 treatment downregulated IL-33-induced genes that encoded for critical signaling pathways of IL-33 activation, including unfolded protein response, TNF signaling *via* NF-κB, mTORC1 signaling, IL-2 and STAT5 signaling, and PI3K and AKT signaling (Fig. 6d).

To confirm the differential gene expression observed in our RNA-seq dataset, we performed qPCR on FACS-sorted peritoneal MCs and found that anti-S8 treatment similarly decreased the expression of a subset of genes induced by IL-33 compared to isotype control mAb treatment (Fig. 6e). Lastly, we examined differential gene expression of peritoneal MCs treated with an isotype control mAb or anti-S8 that were not exposed to IL-33 to evaluate gene expression changes that could be related to Siglec-8-mediated inhibition in non-activated MCs. Interestingly, anti-S8 treatment increased the expression of other genes associated with inhibitory functions, including *Tmem176a*, *Ticam2*, *Tifab*, and *Unc93b*, suggesting Siglec-8-mediated inhibition of MCs may utilize additional inhibitory receptors (Fig. S5E). These data suggest that anti-S8 treatment globally modulates the transcriptome of peritoneal MCs to suppress IL-33-mediated inflammation through MC inhibition.

### Siglec-8 mAb treatment reduces IL-33-mediated neutrophil migration by inhibiting human MCs

**Fig. 7:**
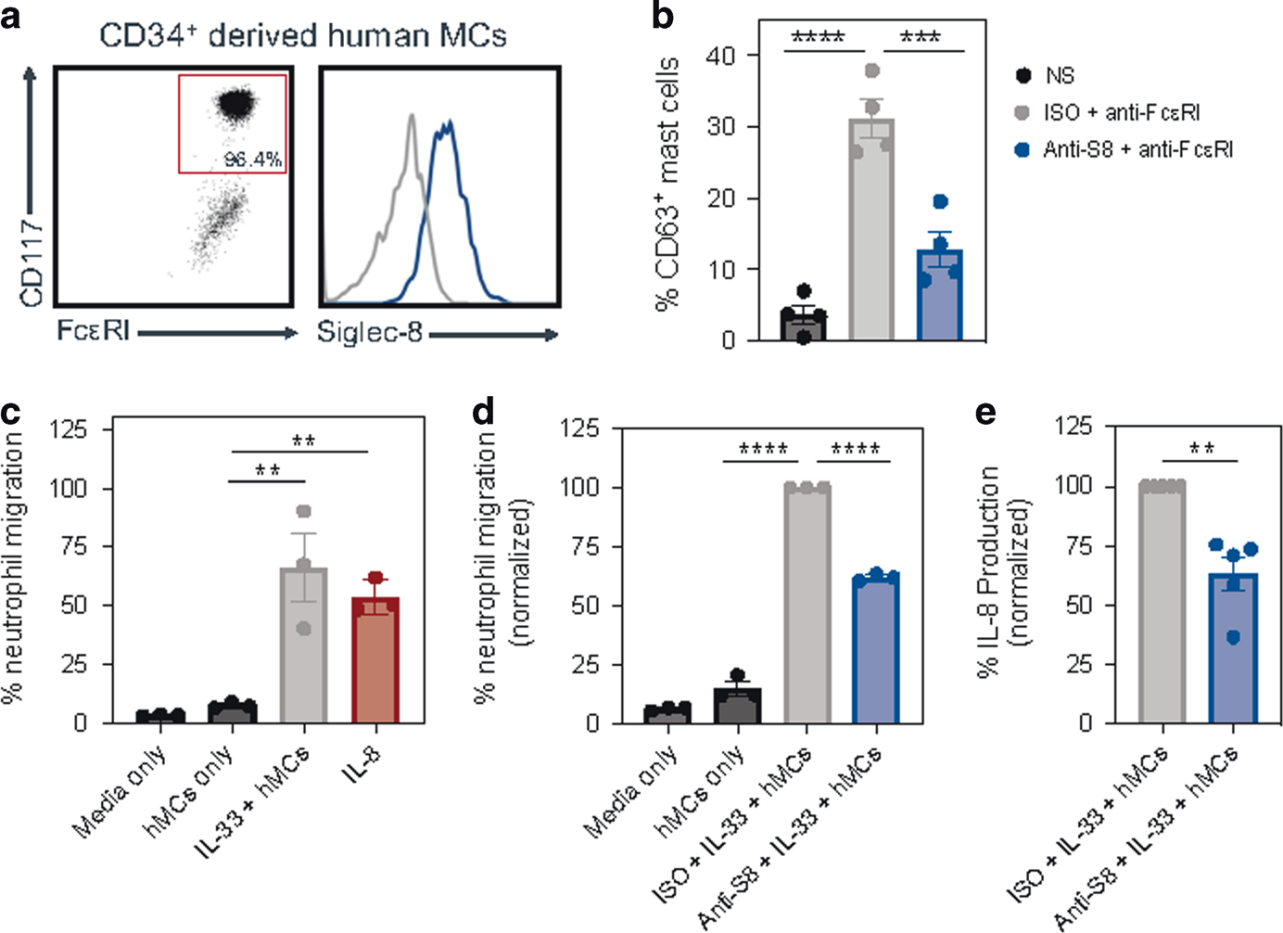
Siglec-8 mAb treatment decreases human neutrophil migration induced by IL-33 activated hMCs. **a** Dot plot showing the percentage of mature hMCs at the end of the differentiation period and the expression of Siglec-8 on mature hMCs (blue) compared to a fluorescence minus one (FMO) negative control (gray). **b** The percentage of CD63 positive hMCs after 20 min of stimulation with anti-FcεRI (5 μg/mL) in the presence of ISO (gray) or anti-S8 (blue) compared to non-stimulated cells (black). **c** The percentage of neutrophil migration induced by media alone (black) or supernatants collected from hMCs alone (dark gray), hMCs stimulated with IL-33 (light gray), or direct IL-8 neutrophil stimulation (red). **d** The percentage of neutrophil migration induced by media alone (black) or supernatants from hMCs alone (dark gray), hMCs stimulated with IL-33 + ISO (light gray), or +anti-S8 (blue). **e** Normalized level of IL-8 in hMC supernatant stimulated with IL-33 + ISO (light gray) or anti-S8 (blue). Data are plotted as mean ± SEM (3–5 human donors/group) and are representative of at least two experiments. For panels **d** and **e**, neutrophil migration and IL-8 levels were normalized to ISO-treated hMCs. *P < 0.05; **P < 0.01; ***P < 0.001; ****P < 0.0001 by one-way ANOVA with Tukey’s multiple-comparisons test or by Mann–Whitney *U* test. ISO isotype control, hMCs human peripheral blood derived mast cells, SEM standard error of the mean.

Since Siglec-8 is natively expressed on human MCs, we sought to extend the relevance of our data from murine models to humans using mature human MCs (hMCs) derived from peripheral blood progenitors.^22,24^ Consistent with the known expression pattern of Siglec-8, fully mature hMCs expressed Siglec-8 (Fig. 7a). To determine whether endogenous Siglec-8 expression levels were sufficient to mediate inhibition in these cells, we activated hMCs through the IgE pathway using an anti-FcεRI antibody in the presence of anti-S8 or an isotype control mAb. Treatment with anti-S8 significantly reduced hMC activation as evidenced by decreased CD63 expression (Fig. 7b), suggesting Siglec-8 is functional in these cells.

To assess the effects of anti-S8 treatment on IL-33-mediated hMC activation, we adapted a human neutrophil migration assay previously shown to be mediated by IL-33-stimulated MCs.^6^ Neutrophil migration toward supernatants from IL-33-activated hMCs was significantly increased compared to supernatants from unstimulated hMCs (Fig. 7c). Directly stimulating neutrophils with the chemoattractant IL-8 significantly increased neutrophil migration to a similar extent as supernatant from IL-33-activated hMCs, suggesting hMCs produce neutrophil chemotactic factors upon IL-33 stimulation (Fig. 7c). Next we assessed if anti-S8 could affect neutrophil migration induced by IL-33 activated hMCs. Supernatant from IL-33-activated hMCs treated with anti-S8 for 6 h showed significantly less neutrophil migration and IL-8 levels compared to supernatant from isotype control-treated hMCs activated with IL-33 (Fig. 7d, e). These data suggest that anti-S8 inhibits IL-33-mediated hMC activation and further highlight MCs as important orchestrators of immune cell recruitment upon stimulation.

## Discussion

MCs are key effector cells that regulate acute and chronic inflammatory responses through the release of preformed and de novo synthesized mediators in response to a diverse array of activating stimuli, including IgE, IgG, cytokines, neuropeptides, and TLR ligands. Aberrant accumulation and activation of MCs is associated with a substantial number of allergic, inflammatory, and proliferative diseases, whereby MCs are thought to be key drivers of inflammation, tissue damage, and remodeling. Siglec-8 has emerged as a promising therapeutic target for allergic and inflammatory diseases due to both its selective expression on MCs and eosinophils and its inhibitory activity. Previous studies showed that anti-S8 mAbs directly inhibit IgE-mediated MC activation in vitro and that this inhibition requires the immunoreceptor tyrosine-based inhibitory motif (ITIM) domain.^24^ To study the activity of anti-Siglec-8 mAb in non-allergic murine models, we used a previously described Siglec-8 tg mouse because of the disparities between human Siglec-8 and its functional paralog in mice, Siglec-F, most notably, the lack of expression of Siglec-F on murine mast cells.^12,15^

Chronic non-allergic airway diseases, including COPD, IPF, and type-2 low asthma, are major respiratory disorders that have limited treatment options. These diseases are associated with increased tissue and BAL neutrophils, whereas chronic allergic airway diseases, such as type-2-high asthma, are characterized by elevated eosinophils. While most current and emerging biologic therapies for asthma specifically target eosinophils and type-2 cytokines, there is a significant unmet need for effective treatments for non-allergic chronic neutrophilic airway diseases.^25,26^ Notably, MCs have been shown to be elevated in patients with chronic non-allergic airway diseases, and mice deficient in MCs have reduced inflammation and tissue remodeling in models of COPD and BLM-induced fibrosis, suggesting MCs contribute to pathogenesis.

Treatment with anti-S8 significantly reduced infiltration of immune cells into the airways induced by CS or BLM, including neutrophils, monocytes, macrophages, and lymphocytes, compared to mice receiving an isotype-matched control mAb. The reduction in immune cell infiltrate was consistent with decreases in cytokines and chemokines that recruit these immune cells in anti-S8 treated mice. The significant decrease in CS and BLM-induced inflammation by anti-S8 treatment was associated with reduced MC numbers and activation, consistent with the inhibitory activity of these mAbs. In addition to reducing airway inflammation, therapeutic anti-S8 treatment decreased histopathologic changes in lung tissue induced by CS, and attenuated BLM-mediated lung fibrosis. These data support previous studies showing that MCs can drive fibrosis and tissue remodeling through the recruitment of immune cells and production of pro-fibrotic mediators, such as TGFβ, CCL2, tryptase, and IL-13.^27^ We did not detect eosinophils in the BAL fluid in the experimental COPD or BLM models. The exact role of eosinophils in these models has not been extensively studied and remains controversial.^28,29^ For example, IL-5-deficient mice that lack eosinophils still develop BLM-mediated fibrosis, suggesting eosinophils do not play a major role in BLM-induced lung injury.^29,30^ Nevertheless, since anti-S8 also has anti-eosinophil activity, we cannot completely exclude the possibility that some of the activity seen with anti-S8 treatment could be due to the reduction of those cells.

To directly evaluate the inhibitory activity of anti-S8 in non-IgE-mediated MC activation, we used a MC-dependent, IL-33-driven model of inflammation. Anti-S8 treatment significantly reduced IL-33-mediated neutrophil, monocyte, and eosinophil infiltration and decreased IL-33-induced cytokines and chemokines by directly inhibiting MCs. Notably, the reduction in IL-33-driven inflammation by anti-S8 was not associated with a decrease in MC numbers which is consistent with an inhibitory, rather than apoptosis-inducing effect of ligating Siglec-8 on MCs.^11,13,15^ Transcriptomic profiling of peritoneal MCs activated by IL-33 and dosed with anti-S8 further confirmed anti-S8-mediated MC inhibition. Anti-S8 treatment significantly and globally modulated IL-33-induced gene expression, highlighting the broad inhibitory activity of Siglec-8. While our data do not identify the exact signaling pathway that leads to anti-S8-mediated MC inhibition, they do confirm that anti-S8 can inhibit non-IgE-mediated MC activation and provide insight into the mechanism of inhibition.

The ability of Siglec-8 to inhibit IL-33-mediated MC activation is intriguing because ITIM-containing receptors are mainly thought to recruit immunoreceptor tyrosine-based activation motif (ITAM)-bearing receptors, such as FcεRI to induce MC inhibition. Previous studies evaluating CD33 (Siglec-3)-mediated MC inhibition showed that antigenic liposomes with CD33 ligand prevented IgE-mediated MC activation by recruiting CD33 to the IgE/FcεRI complex.^31^ Similarly, strategies using bispecific antibodies to co-ligate inhibitory receptors (FcγRIIB or CD300a) with either IgE or FcεRI also demonstrated that coupling the inhibitory and activating receptor is required for IgE-mediated MC inhibition.^32–34^ However, studies evaluating Siglec activity on immune cells other than MCs have shown that Siglecs can inhibit TLR activation in a MyD88-dependent manner.^35,36^ Indeed, Siglec-E in the mouse attenuates LPS/TLR4-mediated activation by decreasing NF-κB activation and the secretion of proinflammatory mediators.^35^ Interestingly, IL-33 also activates NF-κB signaling in MCs *via* the classical MyD88/IRAK/TRAF6 module,^37^ suggesting Siglec-8 may use a similar mechanism as Siglec-E to mediate inhibition. In support of this, transcriptomic profiling of peritoneal MCs that were treated with anti-S8 showed significantly increased expression of genes associated with TLR inhibition and MyD88 signaling, including *Unc93b1, Ticam2*, and *Tifab.*^38–42^ Moreover, TICAM2 and TIFAB have been shown to interact directly with the MyD88/IRAK/TRAF6 complex to regulate TLR signaling.^38,39^ Future studies will focus on further characterizing the intracellular pathways induced by anti-S8 ligation that lead to Siglec-8-mediated inhibition in MCs.

Currently, limited therapeutic options are available in the clinic to treat MC-driven disorders.^2^ Most MC-stabilizing drugs and H1 antagonists have minimal activity against MCs, and many patients become refractory over time, underscoring the importance for new therapeutics.^2^ Anti-IgE monoclonal antibodies, e.g. omalizumab, have been shown to be effective in some allergic diseases, including chronic urticaria, eosinophilic asthma, and nasal polyposis. However, since MCs express multiple activating receptors, and these biologics target only IgE-mediated MC activation, they are likely to be less effective in complex allergic and non-IgE-driven diseases.^2,43,44^ Targeting Siglec-8 with the anti-S8 mAb, AK002, represents a new therapeutic approach that has shown promising effects in early clinical studies in patients with eosinophilic and mast cell-driven disorders. The data reported here further expand on the activity of anti-S8 mAbs for treatment of non-allergic diseases that involve MCs and support the evaluation of AK002 as a therapeutic approach in both allergic and non-allergic inflammatory diseases.

## Methods

### Mice

Siglec-8 tg mice were generated as previously described.^6^ Female and male Siglec-8 tg mice were maintained at Taconic Biosciences (Germantown, NJ, USA) and used at 8–12 weeks of age. All animal experiments were done under an IACUC-approved protocol. For all studies, animals were formally randomized, technicians were blinded to treatment groups.

### Model of cigarette-smoke-induced experimental COPD and assessment of inflammation and lung function

Siglec-8 tg mice (6–8 weeks of age) were simultaneously exposed to CS (twelve 3R4F reference cigarettes (University of Kentucky, Lexington, Ky) twice per day, five times per week for 12 weeks) using a custom-designed and purpose-built nose-only, directed flow inhalation and smoke-exposure system (CH Technologies, Westwood, NJ) housed in a fume and laminar flow hood as previously described.^3,4,45,46^ Each exposure lasted 75 min on week 8, mice were dosed intraperitoneally at 5 mg/kg once a week with either an anti-Siglec-8 mIgG1 mAb (2E2 clone, Allakos, Inc) or isotype-matched control mIgG1 mAb (Biolegend) until week 12. Airway inflammation was assessed by means of differential enumeration of inflammatory cells in BAL fluid.^4,45^ Lungs were perfused, inflated, embedded in paraffin, and sectioned. Histological scoring, airway, vascular, and parenchymal inflammation were assessed by counting the number of inflammatory cells in a minimum of four randomized fields of H&E-stained lung sections as previously described.^47^ To assess lung function, mice were anaesthetized using ketamine (100 mg kg^−1^) and xylazine (10 mg kg^−1^), tracheas cannulated and attached to Buxco® Forced Maneuvers apparatus (DSI, St. Paul, MN, USA) to assess total lung capacity (TLC).^4^ FlexiVent apparatus (FX1 System; SCIREQ, Montreal, Canada) was used to assess FVC and elastance (tidal volume 8 mL kg^−1^, respiratory frequency 450 breaths min^−1^).^4^ All experiments were approved by the University of Newcastle animal ethics committee. Measurements were done without the opportunity for bias.

### Acute model of IL-33-induced peritoneal inflammation

Siglec-8 tg mice (6–10 weeks old) were injected intraperitoneally with PBS or 10–50 ng of recombinant mouse IL-33 (Biolegend) as previously described.^6^ Three hours later cells within the peritoneal cavity were collected by lavage with RPMI and immediately analyzed by flow cytometry. An anti-Siglec-8 mIgG1 mAb (2E2 clone, Allakos, Inc) or isotype-matched control mIgG1 mAb (Biolegend) were administered intraperitoneally at 5 mg/kg 1 h before IL-33 administration.

### Statistics

To determine statistical significance, nonparametric Mann–Whitney *U* test, unpaired two-tailed *t* test, two-tailed *t* test with Holm–Šídák’s posttest, or one-way ANOVA with Tukey’s posttest for multiple comparisons was performed using Prism (GraphPad Software). A *P*-value of 0.05 or less was considered significant.

## Supplementary information

Supplementary Material.
