# Supplementary material for: A monoclonal antibody to Siglec-8 suppresses non-allergic airway inflammation and inhibits IgE-independent mast cell activation
Source: Mucosal Immunol. 2020 Aug 19;14(2):366–76. doi: 10.1038/s41385-020-00336-9 (PMC7946634; doi:10.1038/s41385-020-00336-9)
Supplement: Supplementary file 1 — Supplementary Material. [file 41385_2020_336_MOESM1_ESM.pdf]

**Supplementary Material**

*Enumeration of lung mast cells and degranulation in experimental COPD*

Lungs were perfused, inflated, embedded in paraffin, and sectioned at 3 μm thickness at the end of study on day 84. Slides were stained with chloracetate esterase (Sigma Aldrich) for mast cells (red/brown) and mast cell degranulation (Fig. S1B). Total mast cells and degranulating mast cells were enumerated based on color and morphology in each lung section.

*Acute and chronic models of BLM-induced lung fibrosis and assessments of inflammation and fibrosis*

Acute airway inflammation and fibrosis was induced in Siglec-8 tg mice through a single administration of 1.5U/kg of BLM sulfate (Euroasia's, Batch #20141005) or saline vehicle control *via* the oropharyngeal route as per body weight of the animal as previously described.[1] Mice were harvested on day 7 post-BLM, and inflammation and fibrosis were assessed by a blinded pathologist. An anti-Siglec-8 mIgG1 mAb (2E2 clone, Allakos, Inc) or isotype-matched control mIgG1 mAb (Biolegend) were dosed once intraperitoneally at 5 mg/kg 3 days post-BLM administration. The chronic BLM-mediated lung injury and fibrosis model was induced in Siglec-8 tg mice by subcutaneous injection of 0.1 IU BLM sulfate (Euroasia's, Batch #20141005) or equivalent volume of saline every 2 days through day 29. On day 30, mice were harvested for inflammatory and fibrosis assessments. Anti-S8 or the control mAb were therapeutically dosed intraperitoneally at 5 mg/kg on days 7, 14, 21, and 28 days post the initial BLM administration. BAL was harvested at the indicated time points and stained with toluidine blue to quantify total leukocyte numbers and the remaining cells were used to determine differential counts using a cytospin analyzer. Cytokines and chemokines were analyzed in cell-free BAL supernatant using Luminex analysis (Millipore). Lung fibrosis was assessed by a blinded pathologist using a modified Ashcroft score as previously described[2] on day 7 and 30 in acute and chronic BLM-induced models, respectively. Right lungs were inflated with 10% neutral-buffered formalin, and then placed in a cassette in formalin overnight at room temperature, then transferred to 70% ethanol and stored at room temperature. The fixed segment was processed by standard methodologies, embedded in paraffin, sectioned, and stained with Masson's trichrome. The trichrome-stained sections were used to assess fibrosis via the modified Ashcroft score.[3] Left lungs were flash frozen and used to assess hydroxyproline levels according to the manufacturer's instructions (Cell Bioassays, St. Louis, MO, Hydroxyproline Assay Kit, Catalog #6040). The acute BLM and chronic BLM models were performed at Aragen Biosciences (Morgan Hill, CA) and Murigenics (Vallejo, CA), respectively with technicians blinded to treatment groups.

*RNA-seq and qPCR on isolated peritoneal mast cells*

Siglec-8 tg mice were treated with either an anti-Siglec-8 mIgG1 mAb or isotype control mIgG1 mAb one hour before administration of IL-33. Peritoneal lavages were collected 3 hours after IL-33 administration and MCs from individual mice were FACS-sorted directly into Trizol LS (Invitrogen) and subjected to low-input RNA extraction, library preparation, and RNA-sequencing using the Illumina HiSeq platform. RNAseq data was aligned to the mouse GRCm38.p6 r98 reference and counts were determined using Rsubread version 1.34.7;[4] fold-change and differential expression between treatment groups were computed using DESeq2 version 1.24.0.[5] Gene enrichment analyses were done using the hypergeometric test implemented in the enrich function of the clusterProfiler R package (version 3.12.0).[6] To identify genes induced by IL-33 and/or modulated by anti-S8 treatment, we applied empirically-determined criteria (estimated log2Fold increase in expression greater than 0.4 in the IL-33 modulated condition with a corresponding decrease of 0.4 or more in the IL-33 + anti-S8 condition or *vice versa*, with a *P*-value < 0.05 for the Wald test as implemented in the DESeq2 R package). For confirmatory qPCR analysis, peritoneal MCs were FACS-sorted in RLT lysis buffer (Qiagen) and RNA was extracted according to methods provided by the manufacturer (Qiagen). cDNA was synthesized according to the manufacturer's protocol (Thermo Fisher Scientific), and qPCR was performed using SYBR green (Thermo Fisher Scientific) and predesigned gene-specific primers for *B2m, Gata2, Hdc, Nfkb1, Nfkbiz, Tnf, Tpsab1*, *Trib3, Tmem176a, Ticam2, Tifab, and Unc93b1* (IDT). Relative gene expression was determined by normalizing the Ct for each gene by sorted MC number and calculating the fold-change based on the normalized Ct values in the PBS-treated group.

*MCPT-1 and MCPT-4 quantification*

mMCPT1 and mMCPT4 levels were quantified in the lungs of bleomycin challenged or sham mice by harvesting the lungs on day 7, followed by overnight culture in RPMI and collection of supernatants. Serum was collected on day 84 of the experimental COPD study. Supernatants and serum were analyzed for mMCPT1 (ThermoFisher, Cat # 88-7503-22) and mMCPT4 (LSBio, LS-F55860-1) by ELISA according to the manufacturer's instructions.

*Peritoneal mast cell in vitro stimulation and cytokine/chemokine analysis*

Peritoneal MCs were isolated by lavage, followed by culture in RPMI supplemented with recombinant stem cell factor and IL-3 (R&D systems) until MCs were highly enriched. Purity of peritoneal MC cultures was determined to be >95% by flow cytometry before in vitro stimulation experiments. Approximately $2.5\times10^4$ peritoneal MCs were stimulated with recombinant mouse IL-33 overnight at the indicated concentrations, followed by collection of cell-free supernatant for cytokine analysis. Levels of cytokines and chemokines in the serum, supernatant, and peritoneal lavage were determined by multiplex analysis (MSD).

*Human peripheral blood derived MCs*

Peripheral blood cells were isolated from residual cells in the leukocyte reduction chamber (TrimaAccel). Cells were eluted by gravity, and RBCs were lysed using 1X lysis buffer (BioLegend). CD34+ progenitor cells were isolated using the CD34 MicroBead Kit UltraPure human kit (Miltenyi Biotec) and cultured as previously described (Saito, H et al. 2006). After 7 weeks in culture, cells were maintained IMDM supplemented with 5% FBS, 55 μM β-Mercaptoethanol, 100ng/mL SCF, and 50ng/mL IL-6.

*Human neutrophil migration assay*

Migration studies were performed using the Transwell-96 well permeable support with 5.0 μm pore polycarbonate membrane (Corning Costar). Neutrophils were isolated from whole blood using the EasySep direct neutrophil isolation kit (STEMCELL Inc) and purity was >95%. Cells were resuspended to $5\times10^6$ cells/ml and 75 μl of the cell suspension was added to the upper chambers. The lower chambers were loaded with supernatants isolated from isotype control mIgG1 mAb (Biolegend) or anti-Siglec-8 mIgG1 mAb (m2E2 clone, Allakos Inc) treated (5 μg/mL) mast cells that were stimulated with either vehicle or IL-33 (10 ng/mL) for 6h. Supernatants were analyzed for cytokines using MSD as described above. As a positive control, IL-8 (100 ng/ml) was also added in a separate well. After 1 hour of culture at 37°C, chemotaxis was determined by comparing the ratio of migrated cells to the total number of input cells as assessed by flow cytometry. IgE-mediated hMC activation was induced by stimulating with anti-FcεRI (10 μg/mL, CRA-1 clone, BD Biosciences) for 20 minutes at 37°C in the presence of an isotype control or anti-S8 mAb (5 μg/mL) and activation was assessed by flow cytometry using CD63 expression.

*Flow cytometric analyses of blood and tissue*

Approximately $1–5\times10^6$ cells were preincubated with CD16/32 antibody to block nonspecific binding. Cells were then incubated at 4°C for 10 minutes with staining antibody panels, washed, and fixed in 2% paraformaldehyde. Data acquisition was performed using a NovoCyte flow cytometer (Acea Biosciences) and FlowJo (San Diego, CA) was used for data analysis. The following antibodies were purchased from eBioscience or Biolegend (clone indicated in brackets): CD4-BV650 (RM4-5), CD8-BV650 (53-6.7), CD11b-BV605 (M1/70), CD45-BV785 (30-F11), CD117-SB436 (2B8), F4/80-PECy7 (BM8), Ly6G-BV510 (1A8), Ly6C-APCCy7 (HK1.4), SiglecF-PE (S17007L), FcER1-FITC (MAR-1), and Siglec-8 AF647 (1H10 clone, Allakos, Inc.). Gating strategy for cells from mouse tissues was as follows: mast cells: $7AAD^-$ $CD45^+$ $F480^-$ $CD11b^-$ $IgER^+CD117^+$; Eosinophils: $7AAD^-$ $CD45^+$ $SSC^{Hi}$ Siglec-$F^+$; Neutrophils: $7AAD^-$ $CD45^+$ $Ly6C^-$ $Ly6G^+$; Monocytes: $7AAD^-$ $CD45^+$ $Ly6G^-$ $Ly6C^{hi}$, Macrophages $7AAD^-$ $CD45^+$ $F4/80^{hi}$.

**References**

1. Walters, D. M. & Kleeberger, S. R. Mouse models of bleomycin-induced pulmonary fibrosis. *Curr. Protoc. Pharmacol.* **Chapter 5**, Unit 5.46 (2008).

2. Hubner, R.-H. *et al.* Standardized quantification of pulmonary fibrosis in histological samples. *Biotechniques* **44**, 507-511,514-517 (2008).

3. Jarnicki, A. G. *et al.* The inhibitor of semicarbazide-sensitive amine oxidase, PXS-4728A, ameliorates key features of chronic obstructive pulmonary disease in a mouse model. *Br. J. Pharmacol.* **173**, 3161–3175 (2016).

4. Liao, Y., Smyth, G. K. & Shi, W. The R package Rsubread is easier, faster, cheaper and better for alignment and quantification of RNA sequencing reads. *Nucleic Acids Res.* **47**, e47 (2019).

5. Love, M. I., Huber, W. & Anders, S. Moderated estimation of fold change and dispersion for RNA-seq data with DESeq2. *Genome Biol.* **15**, 550 (2014).

6. Yu, G., Wang, L.-G., Han, Y. & He, Q.-Y. clusterProfiler: an R package for comparing biological themes among gene clusters. *OMICS* **16**, 284–287 (2012).

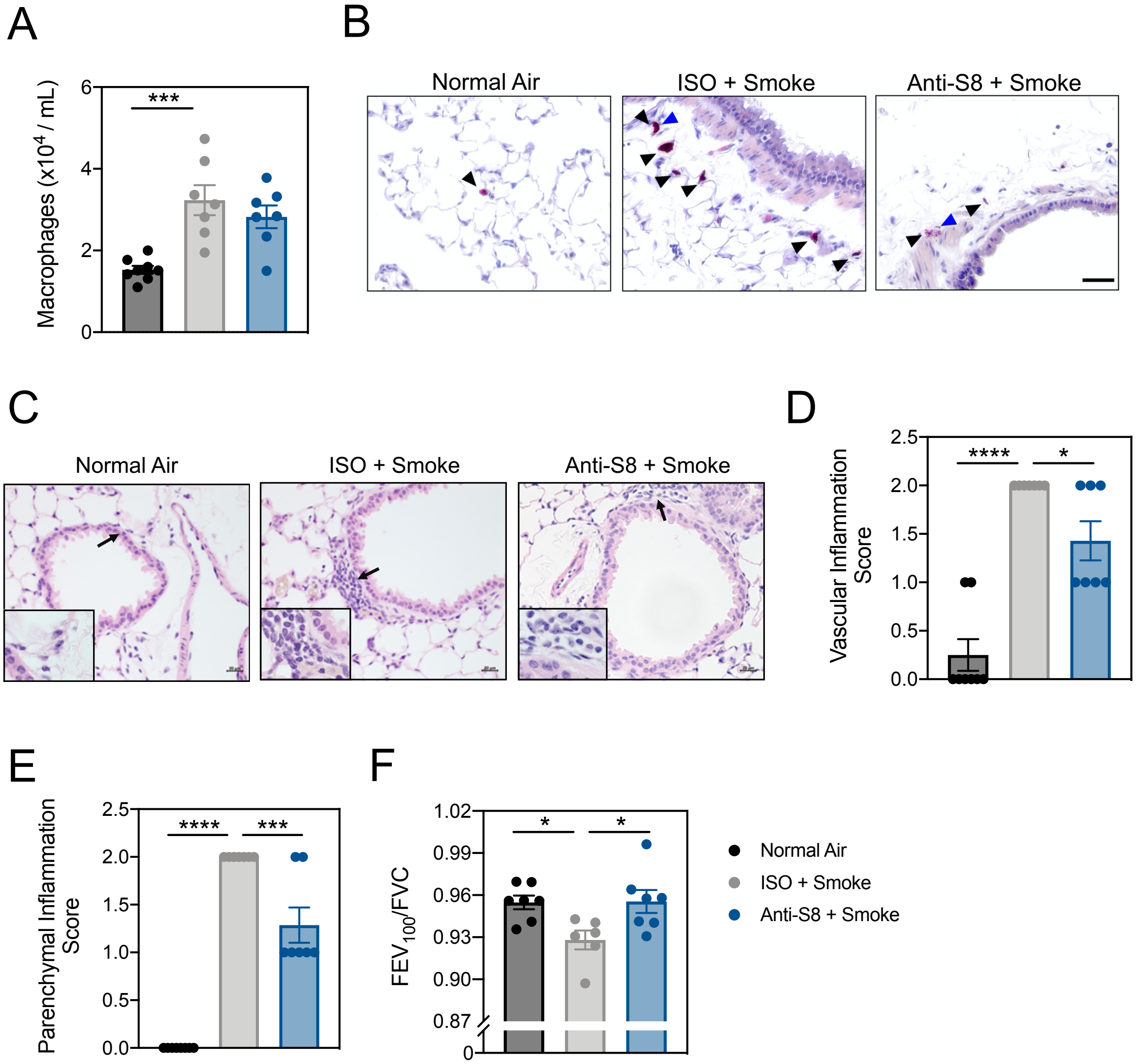


**Fig. S1: Therapeutic treatment with anti-S8 reduces lung inflammation and improves lung function in experimental COPD.** (A) Macrophages in BAL fluid in mice exposed to normal air (black), ISO + smoke (gray) or anti-S8 + smoke (blue). (B) Representative images of lung mast cells identified by tryptase staining. Intact granule and degranulating MCs are noted by black and blue arrows, respectively. (C) Representative images of lung histopathology with black arrows highlighting inflammation. (D and E) Histopathology scores specifically in the vascular and parenchymal regions. (F) Lung function was assessed in terms of $FEV_{100}$/FVC in in mice exposed to normal air (black), Isotype control mAb + smoke (gray) or Anti-S8 + smoke (blue). Data are plotted as mean +/- SEM (7-8 mice/group) * $P < 0.05$; *** $P < 0.001$; **** $P < 0.0001$ by 1-way ANOVA with Tukey's multiple-comparisons test. BAL, bronchoalveolar lavage; $FEV_{100}$/FVC, forced expiratory volume in 100 msec/forced vital capacity ratio; ISO, isotype control; SEM, standard error of the mean.

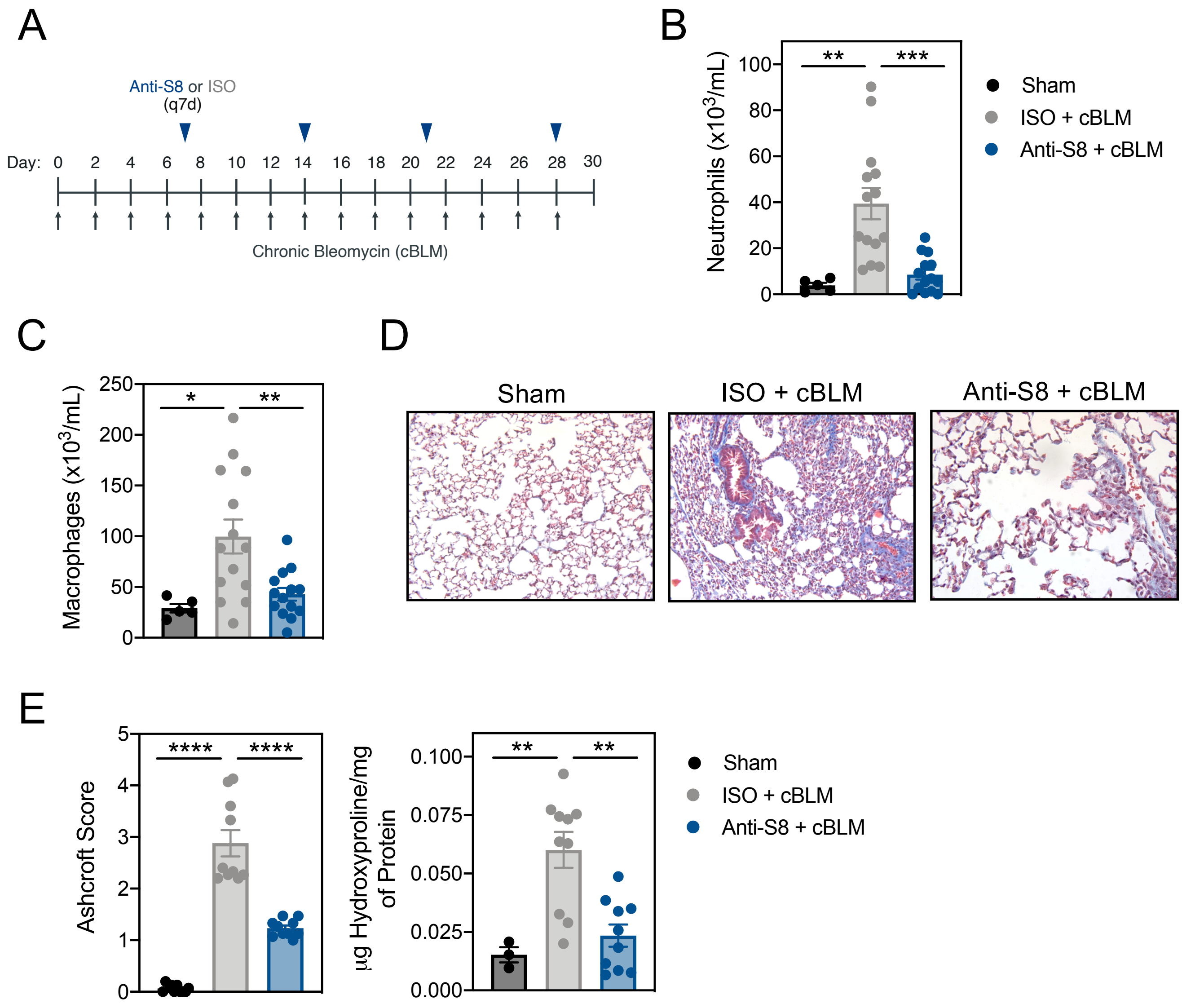


**Fig. S2: Therapeutic treatment with anti-S8 reduces lung inflammation and fibrosis in a chronic BLM-induced lung injury model.** (A) Chronic BLM-induced lung fibrosis model. (B and C) Neutrophils and macrophages in the BAL fluid of sham (black), ISO + chronic BLM (gray) or anti-S8 + chronic BLM (blue) treated mice. (D) Representative Masson's trichrome staining images and (E) Ashcroft score of lung fibrosis and lung hydroxyproline levels in sham (black), ISO + chronic BLM (gray) or anti-S8 + chronic BLM (blue) treated mice. Data are plotted as mean +/- SEM (10-14 mice/group) and are representative of at least 3 experiments. *$P < 0.05$; ** $P < 0.01$; *** $P < 0.001$; **** $P < 0.0001$ by 1-way ANOVA with Tukey's multiple-comparisons test. BAL, bronchoalveolar; cBLM, chronic bleomycin; ISO, isotype control; SEM, standard error of the mean.

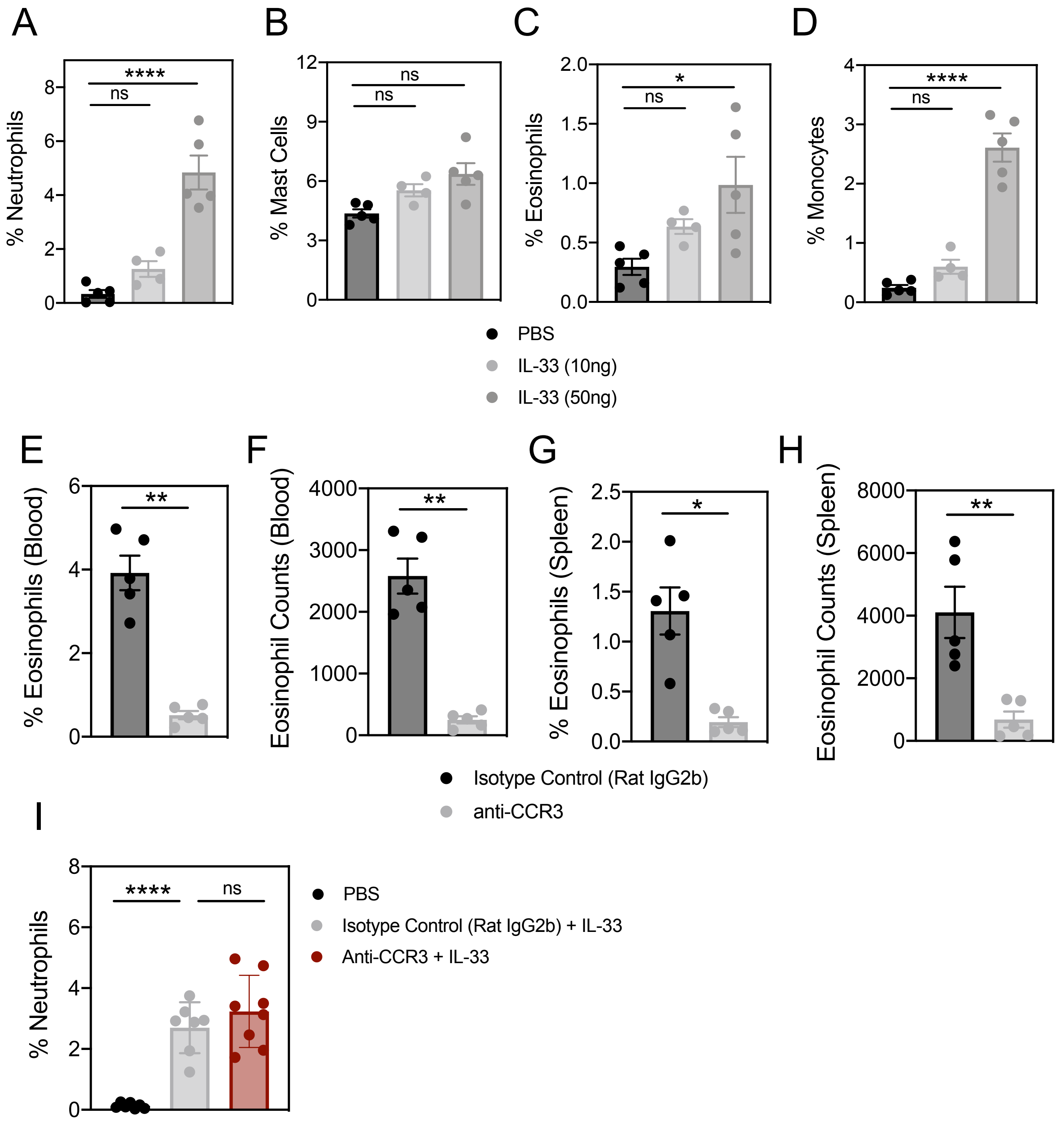


**Fig. S3: IL-33 administration induces leukocyte infiltration in the peritoneal cavity that is not mediated by eosinophils.** Percentage of (A) neutrophils, (B) mast cells, (C) eosinophils, and (D) monocytes in the peritoneal cavity in mice treated with PBS (black), 10 ng IL-33, or 50 ng IL-33 for 3 hours. (E-H) Percentage and counts of blood/mL and spleen eosinophils in mice that were administered isotype control antibody (black) or anti-CCR3 antibody (gray) at 5 mg/kg. (I) The percentage of neutrophils in the peritoneal cavity in mice treated with PBS (black), Isotype control (rat IgG2b) + IL-33 (50 ng) (gray), or anti-CCR3 + IL-33 (50 ng) (red). The percentage of immune cells was derived from CD45+ viable cell population. Data are plotted as mean +/- SEM (n=5-8/group). **P* < 0.05; ** *P* < 0.01; *** *P* < 0.001; **** *P* < 0.0001 by 1-way ANOVA with Tukey's multiple-comparisons test or by Mann-Whitney U test. ns, not significant.

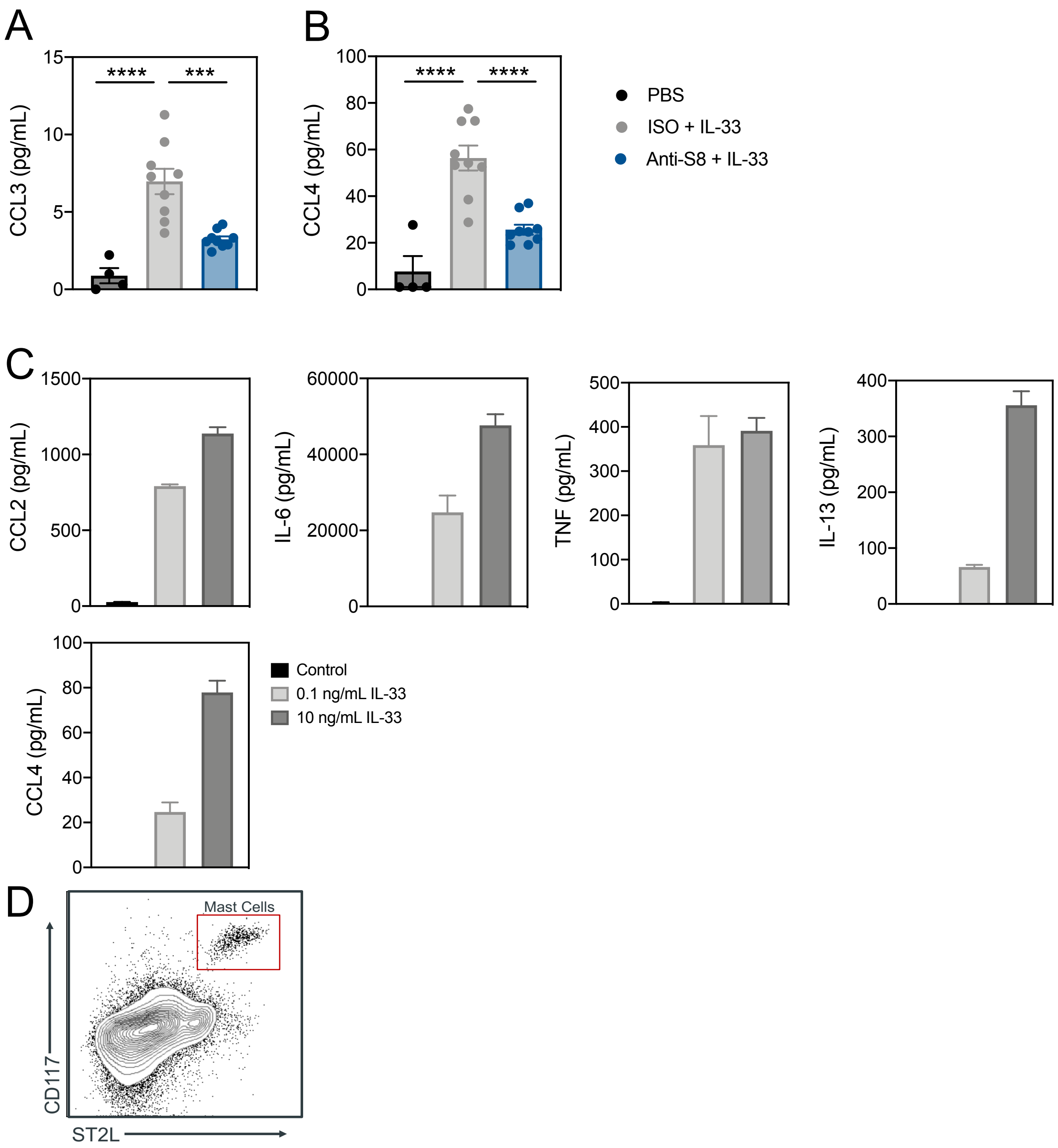


**Fig. S4: Anti-S8 treatment decreases cytokines induced by IL-33 in vivo and peritoneal MCs produce similar mediators in vitro after IL-33 stimulation.** (A and B) Levels of MIP-1a and MIP-1b in the peritoneal cavity of mice treated with PBS (black), ISO + IL-33 (gray) or anti-S8 + IL-33 (blue). (C) Cytokine and chemokine levels produced by in vitro cultured peritoneal MCs ($5x10^4$ cells/well) after overnight stimulation with 0.1 ng/mL IL-33 (light gray), 10 ng/mL IL-33 (dark gray), or unstimulated control (black). (D) Expression of ST2L on MCs from freshly isolated peritoneal lavage. Data are plotted as mean +/- SEM (9-10 mice/group) and are representative of at least 4 experiments in A and B. *** $P < 0.001$; **** $P < 0.0001$ by 1-way ANOVA with Tukey's multiple-comparisons test. ISO, isotype control; MC, mast cell; PBS, phosphate buffered saline; SEM, standard error of the mean.

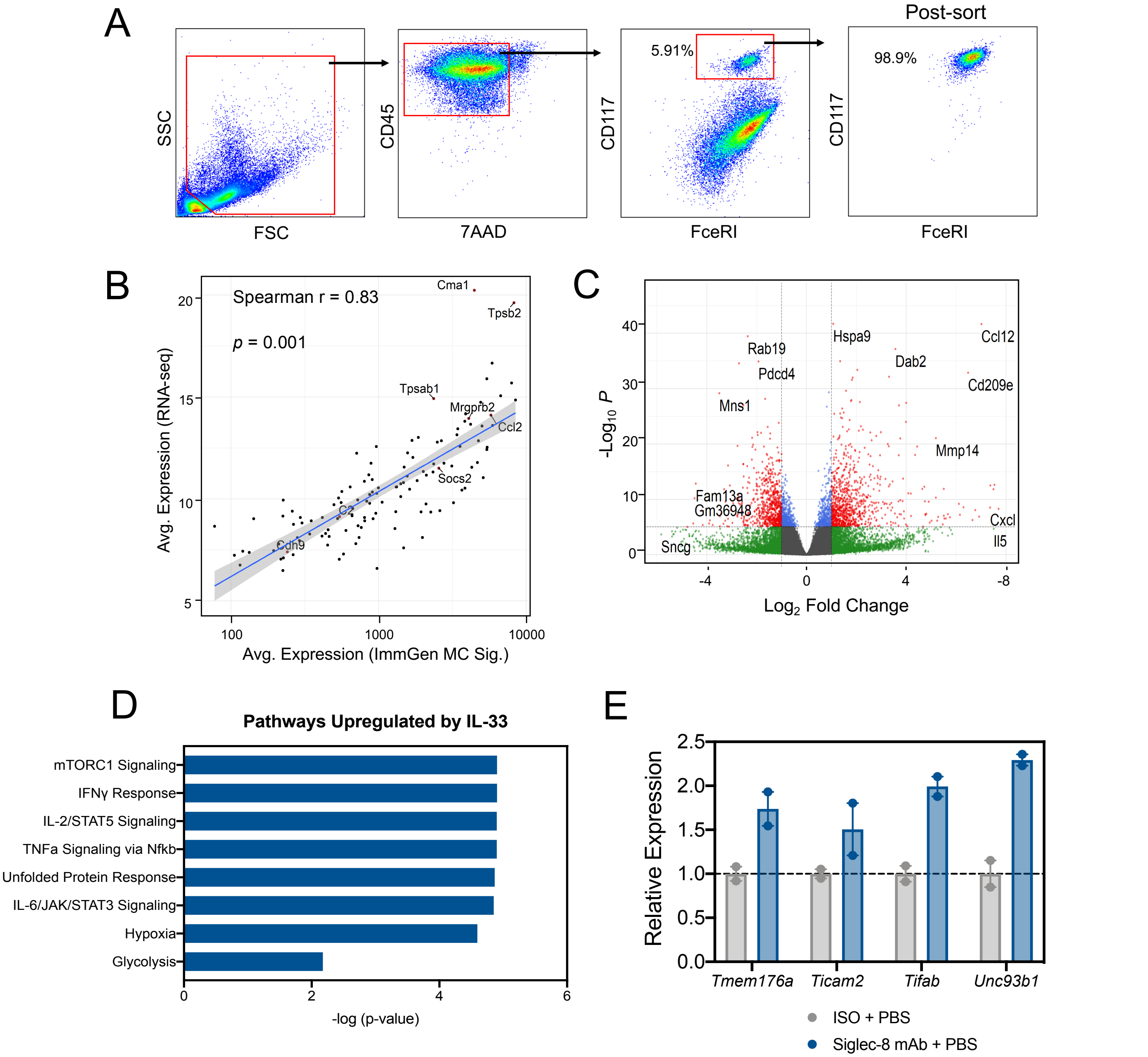


**Fig. S5: Transcriptional profile of peritoneal MCs after IL-33 administration in vivo**. (A) FACS-sorting gating strategy for isolating peritoneal MCs from individual mice based on CD117 and FceRI expression. (B) Correlation between genes identified in our RNA-seq data with the previously published transcriptional signature of tissue-resident MCs (ImmGen). (C) Volcano plot comparing differentially expressed genes among mice treated with PBS or IL-33 for 3 hours. (D) Hallmark gene enrichment analysis highlighting pathways that are significantly associated with genes that are increased in IL-33-activated MCs compared to PBS treated. (E) Confirmatory qPCR analysis of FACS-sorted peritoneal MCs treated with PBS + ISO (gray) or anti-S8 (blue). Dashed line represents normalized gene expression of PBS control peritoneal MCs. For panel E, data are plotted as mean +/- SEM (n=2/group; 3 mice pooled/individual group). FACS, fluorescence activated cell sorter; GSEA, gene set enrichment analysis; MC, mast cell; PBS, phosphate bufferred saline.
